# Humor and Attachment: Exploring the Relationships between Insecure Attachment and the Comic Styles

**DOI:** 10.3390/ejihpe13010012

**Published:** 2023-01-12

**Authors:** Alberto Dionigi, Mirko Duradoni, Laura Vagnoli

**Affiliations:** 1 Studio Psi.Co., 47841 Cattolica, Italy; 2 Department of Education, Languages, Interculture, Literatures, and Psychology, University of Florence, 50135 Florence, Italy; 3 Meyer Children’s Hospital IRCCS, Pediatric Psychology, 50139 Florence, Italy

**Keywords:** comic styles, humor, attachment styles, anxious attachment, avoidant attachment

## Abstract

In this study, the relationship between individuals’ insecure attachment styles and eight comic styles was explored. A sample of 636 Italian adults (206 males, 428 females, 2 non-binary), aged 18 to 81 years (M = 41.44; DS = 13.44) completed an online survey to investigate the relationship between insecure attachment styles, namely anxious and avoidant, and the eight comic styles, clustered into lighter style (fun, benevolent humor, wit, nonsense) and darker style (irony, satire, sarcasm, cynicism). The findings of this research indicated the lighter and darker styles were differently related to the anxious and avoidant styles. The anxious attachment was negatively related to both benevolent humor and wit and positively with irony. The avoidant style was positively associated with nonsense and sarcasm, while no other relationship emerged. This research indicated that attachment orientations are associated with individual differences in the detailed differentiation of humor-related styles.

## 1. Introduction

Attachment theory provides psychological, evolutionary, and ethological frameworks for understanding human development [1]. According to this theory, humans are born with an innate need to form a close emotional bond (the attachment behavioral system) with a caregiver that develops in the first period of life, approximately in the first 12 months of a child’s life [2]. The way the attachment develops depends on a variety of parameters that are gradually shaped by interpersonal interactions with attachment figures [3] resulting in specific differences in the way each person behaves. Attachment represents a specific relationship between the infant and the caregiver, usually the mother, who is perceived to be a source of comfort and security. It develops in an emotional bond leading to a systematic pattern with relative expectations and emotions when in danger that reflects diverse attachment orientations [4]. The quality of that bond is affected by the way the caregiver responds to the infant’s needs, and this results in different attachment patterns: secure or insecure.

Having a responsive and available caregiver results in the development of a secure bond. People who develop a secure attachment have a positive sense of self (that is reflected in low anxiety) and a positive perception of others (low avoidance). They tend to be more socially self-confident and do not fear rejection from others, feeling comfortable when emotionally close to others [5]. Conversely, in case of damaging interactions with inconsistent, unresponsive, and/or unavailable caregivers, an insecure bond will develop, characterized by negative internal working models of the self and others [6]. Adult insecure attachment styles are suggested to be clustered into two main dimensions: anxious and avoidant [5,7].

The avoidance attachment dimension is characterized by a negative view of others, reflecting a negative expectation about the likelihood of receiving support and comfort from others, discomfort with closeness in relationships, and the suppression of attachment-related needs [1]. People with avoidant attachment experience discomfort regarding closeness and dependence on others; they are less prone to seek support from others and tend to deactivate their attachment system [1,8]. Conversely, the anxious attachment style is characterized by a negative view of the self, searching for closeness with others based on an evaluation of personal worth, and increased negative affect. Individuals with an anxious attachment style tend to fear rejection and abandonment [5]. Attachment styles affect self-identity and intimate relationships and form vulnerability to psychopathology [9]. Research has largely demonstrated that humor may have important effects on personal and relational well-being. Humor has an important role in creating and maintaining interpersonal relationships, and the production of a positive form of humor is facilitated by a sense of security and safety [10]. Moreover, a large amount of research has demonstrated that insecure attachment dimensions are correlated with lower well-being, poor psychological and marital satisfaction, maladaptive coping strategies, and negative responses to stressful conditions [9,11,12]. As attachment styles reflect the willingness of people to create emotional bonds, this can be, in turn, related to the individual’s kind of humor appreciation and production. Previous research has shown that secure attachments are related to positive forms of humor, addressed to favoring emotional bonding, while negative forms of humor are related to insecure attachment styles as underlined by an individual disinterest in creating emotional bonds [13]. To date, the studies conducted in this field have not examined the associations between attachment patterns and specific categories of humor; therefore, a deeper understanding of the relationships between attachment styles and a narrower categorization of humor styles may be of interest for better understanding the connection between these two constructs.

### 1.1. Humor and Attachment

Prior research has shown that humor may have important effects on personal and relational well-being [14]. As attachment has been linked to poor wellbeing, it is reasonable to determine whether the attachment styles relate to different ways in which humor is expressed. Several studies have shed light on this connection.

The majority of the studies conducted to evaluate the relationships between humor an attachment have used the Humor Styles Questionnaire (HSQ). The HSQ incorporates positive and negative uses of humor, divided into four subscales: two benevolent and two maladaptive. Positive humor styles include affiliative, used to improve relationships and make others laugh, and self-enhancing, which involves using humor as a coping strategy. Negative humor styles include aggressive, which is detrimental to others, and self-defeating humor, detrimental to the self. Many studies have combined the subscales to create adaptive/maladaptive humor styles; however, the common aspect is a conceptualization of positive and negative humor.

Maladaptive humor was found to mediate the relationship between early maladaptive schema domains and depression, proving how humor may interact between internalized schemas and mental health [15]. When the role of parental emotional bonds, adult attachment orientations, and maladaptive humor styles in emotion regulation was explored, findings showed that both adult attachment anxiety and attachment avoidance positively predicted the use of maladaptive humor styles [16].

A recent study revealed a negative trend between avoidant attachment and benevolence, while a positive trend emerged with the detrimental humor styles [13]. Further, a negative correlation emerged between an avoidant attachment style and affiliative humor [17,18,19]. Avoidant individuals exhibit a sense of discomfort with intimacy and closeness, and they are less likely to amuse others as this would lead to enhancing closeness, resulting in avoiding excessive intimacy. Avoidant attachment also reflects a higher use of self-defeating styles of humor and a minor presence of humorous interactions in close relationships [19,20,21]. Moreover, aggressive humor was positively related to attachment avoidance [18,22]. In this case, aggressive humor may be a way to distance others and at the same time allow avoidant individuals to live with and among others.

The anxious attachment was positively associated with detrimental humor styles and a minor tendency to use affiliative humor styles [13]. Results revealed that attachment anxiety was negatively related to affiliative humor, supporting the position that a sense of security underlies affiliative humor use [18,22]. A self-enhancing humor style was found to be negatively related to the anxiety dimensions [19] and, in several studies, the anxious attachment was found to be related to a lower tendency to generate humor and less use of self-enhancing and coping humor [19,20,21]. This result was also supported by new findings that indicated how self-defeating humor predicted the anxious attachment style in friendships [17]. This can be explained by the fact that humor has been found to correlate with trait anxiety, low intimacy, and low self-esteem [23,24]. These individuals may use this style of humor to try to enhance their relationships at their own expense [17]. In general, higher levels of attachment insecurity predicted higher use of maladaptive humor, which in turn predicted higher distress levels [25].

The studies conducted so far have mainly assessed how people with different attachment orientations use humor with diverse functions, but it still remains a prominent field of research as no association has been conducted with specific kinds of humor (e.g., irony, sarcasm, etc.). In recent years, a new model of humor emerged, focused on a list of eight lower-level styles, namely the Comic Style Markers (CSM) [26,27]. The eight comic styles can be differentiated as lighter or darker styles of humor and include fun, humor, nonsense, wit, irony, satire, sarcasm, and cynicism [27]. The four lighter styles, which relate to benign and social affect, behaviors, cognitions, and goals, are (1) fun, aimed at spreading good mood and good companionship; (2) humor aimed at arousing the sympathy and shortcomings of fellow humans, discovering discrepancies in everyday experiences and treating them humorously and benevolently; (3) nonsense based on playing with incongruities and ridiculousness with no specific purpose; and (4) wit, referred to as the ability to create clever connections between ideas and thoughts. Conversely, the darker styles that lack this benevolent effect and are mostly based on mockery and ridicule are (1) irony, creating a mutual sense of superiority toward others by saying things that are untrue to their intent and contain the opposite of what is intended and understood by insiders; (2) satire, deprecating the bad and foolish, using humor to criticize and correct shortcomings, misconduct, and moral wrongdoings to improve the world and correct fellow humans; (3) sarcasm, grounded on being critical of others and conveying contempt; and (4) cynicism, exhibiting a negative and destructive attitude aimed at devaluing commonly recognized values.

Conceptually, the humor and comic styles are different in terms of the derivation of the constructs, the degree of abstraction, and the scope [25]. However, some studies have shown that the comic styles show specific correlations with the humor styles [25,28,29]. Specifically, some styles seem to be similar, although not conceptually interchangeable: fun and affiliative share similarities as they are mainly addressed at identifying humor production in group settings. The nature of benevolent is not dissimilar from self-enhancing humor, as they both share a humorous outlook on life. Finally, both sarcasm and aggressive humor are based on being critical of others [26].

To date, no research has investigated the relationship between attachment and specific categories of humor; therefore, the main aim of this research was to investigate how the insecure attachment styles are related to the eight comic styles.

### 1.2. Aim of the Study

The present study was developed to finely analyze the relationship between the eight comic styles and two aspects of insecure attachment, namely anxious and avoidant, based on Ainsworth’s infant attachment styles literature.

Considering previous research [19,21,22,30], we expected that the lighter styles would be negatively related to the insecure attachment styles, while the darker styles would be positively related.

The specific hypotheses of this cross-sectional study were as follows:

1. Anxious attachment showed negative correlations with affiliative humor, whose purpose is to strengthen ties between individuals. Therefore, we expect negative trends with benevolent humor. Moreover, wit reflects the ability to create clever connections between ideas and thoughts, but anxious individuals may be ashamed, so we also expected a negative relationship.

Due to the fear of rejection, for individuals with insecure attachment, we expect no relationship with fun, the comic style aimed at spreading good mood and good companionship. Finally, anxiety will be positively related to irony due to its ambivalent content that may be used as a way to express one’s refusal without revealing one’s feelings and therefore being more secure. Other relationships are tested exploratively.

2. Avoidant attachment would indicate a negative association with both light and dark style, namely nonsense and sarcasm. We expect these relationships as these comic styles may safely express one’s emotionality without directly being exposed. Again, we expect no relationship with fun, as avoidant individuals inhibit their emotions and distance others. Other relationships will be tested exploratively.

## 2. Materials and Methods

### 2.1. Participants

Data were collected from 636 Italian participants (206 males, 428 females, 2 non-binary), aged 18 to 81 years (M = 41.44; DS = 13.44). The participants were well-educated adults (3.5% had a lower secondary school diploma, 35.5% had an upper secondary school diploma, 36.9% had a university degree, 17.5% had a master’s degree, and 6 had a doctorate). In terms of marital status, 248 (39.0%) were not married, 331 (52.0%) were married or cohabiting, 51 (8.0%) were divorced, and 6 (0.9%) were widowed.

### 2.2. Measures

The CSM [27] is a self-report questionnaire in which participants rate the extent to which 48 statements apply to the way they typically express humor on a 7-point Likert scale from 1 (strongly disagree) to 7 (strongly agree). Total scores correspond to the mean of the 6 items, with higher scores corresponding to greater use of that specific comic style. This study used the Italian version [31]. The 8 scales indicated good to acceptable reliabilities (ω: fun = 0.84; humor = 0.74; nonsense = 0.86; wit = 0.82; irony = 0.75; satire = 0.80; sarcasm = 0.74; cynicism = 0.82).

The attachment was measured using the Experiences in Close Relationships—Short Form (ECR-S) [32]. The ECR-S is a 12-item measure (6 for each style) based on Ainsworth’s infant attachment styles literature, which assesses adult attachment by focusing on the overall experience of their close relationships. Participants are required to rate the statements on a 7-point Likert scale from 1 (strongly disagree) to 7 (strongly agree). Total scores correspond to the mean of the 6 items, with higher scores representing higher dimensions of attachment insecurity. The anxious (ω = 0.71) and the avoidant attachment (ω = 0.83) scales were internally consistent.

### 2.3. Procedure

The research design was cross-sectional, and data were collected utilizing an online survey, whose link was posted on social media and sent by mailing lists. The survey also included a participation agreement and a description of the study’s purpose. The study was performed under local, ethical guidelines, and all participants were guaranteed anonymity. The inclusion criteria were being aged 18 years or older and Italian citizens.

### 2.4. Statistical Analysis

All data were analyzed using SPSS 25.0 (IBM Inc., New York, NY, USA). As a first step, we performed the power analysis to establish an adequate sample size. We relied on G*Power software to accomplish this procedure [33,34]. An essential aspect of experimental design is power analysis, which allows researchers to determine the recommended sample size and detect an effect of a given size with a given degree of confidence. The analysis showed that in our case (i.e., linear multiple regression analysis), a sample size of 213 individuals would be enough to ensure a statistical power of 0.90, assuming even a very small effect size (f^2^ = 0.05) and a significance level of 0.05. The correlations between gender, age, CSM, and ECR-S were analyzed using Pearson correlation coefficients. For each Pearson correlation, we assessed the variables’ normality (asymmetry and kurtosis values), homoscedasticity, and linearity. Hierarchical regressions of the two attachment styles with the eight comic styles were conducted after checking for potential multicollinearity issues.

## 3. Results

### 3.1. Intercollations among the Eight Comic Styles

Table 1 shows the intercorrelations among the eight comic styles. All intercorrelations among the styles were positive, ranging from 0.31 (cynicism and fun) to 0.63 (sarcasm and cynicism). The darker styles showed higher intercorrelations with each other compared to the lighter styles.

### 3.2. Relationship between Demographics, Humor, and Attachment

The insecure attachment styles were correlated with the eight comic styles, as shown in Table 2. Our sample showed specific differences concerning the relationships among the variables.

Table 2 shows that both attachment styles correlated positively with three darker styles, namely irony, sarcasm, and cynicism, except from satire, which showed zero-order relationships. Differences emerged concerning lighter styles: anxious attachment was positively related to fun, while avoidant attachment correlated positively with nonsense. No other relationships emerged.

### 3.3. Associations between the Comic Styles and Attachment

To test the relationships between each comic style and insecure attachment styles, a hierarchical regression controlled for age and gender was employed. Statistical assumptions of the model, such as homoscedasticity, multivariate normality, and absence of multicollinearity, were all met. The general results of the multiple linear regression analyses are summarized in Table 3.

The regression analyses showed that the light and dark styles were differently related to the anxious and avoidant styles. The anxious attachment was negatively associated to both benevolent humor and wit, while irony was positively related to this style (4% explained variance). The avoidant style emerged to be positively associated to both nonsense and sarcasm (4% explained variance), while no other relationship emerged.

## 4. Discussion

In this study, we investigated cross-sectionally how specific categories of humor, explicitly the comic styles, are related to insecure attachment, namely anxious and avoidant. We also investigated the unique role of the relationship between the two attachment styles and the different comic styles, beyond the role of gender and age. Overall, the findings supported our hypotheses that attachment orientations are associated with individual differences in the sense of humor.

In particular, the results showed that irony was positively associated while benevolent humor and wit were negatively reflected by anxious attachment. These results are in line with our expectations. Previous research has indicated that anxious attachment was negatively related to affiliative and self-enhancing humor [13]. Individuals with an anxious attachment style may face difficulty in appreciating humor due to their serious attitude toward relationships inhibiting playfulness, including the playful attitude that perceives opportunities for humor [22]. According to the general theoretical assumptions, individuals with anxious attachment fear rejection and abandonment, and they may inhibit their use of more benevolent forms of humor. Within the comic style framework, benevolent humor represents a way to treat human weaknesses and wrongdoings compassionately, accept imperfections, and smile at adversity, which reflects a cheerful mood and a serene and humorous outlook on life that can be similar in purpose to self-enhancing humor [28]. Cann et al. [19] found that a self-enhancing humor style was negatively related to the anxiety dimensions, and previous research also indicated that self-enhancing humor may lead to greater relationship satisfaction [35], while individuals with both anxious and avoidant attachment report lower levels of relationship satisfaction [36]. We can assume that anxiously attached people because of their feeling of not being properly appreciated and loved are more insecure in their relationships, and they are less likely to use a benevolent kind of humor that is strictly related to having a cheerful outlook on life.

Wit reflects the ability to create clever connections between ideas and thoughts with the desire to be brilliant and also seemed to reflect cognitive ability [27]. Anxious individuals are insecure and fear being rejected, and this is consistent with the negative view of the self that anxious people are presumed to have. Therefore, they disclose less compared to secure people, perhaps because they feared rejection [37], and this can lead to inhibition from saying comments that can capture others’ attention. Moreover, wit represents a form of humor that reflects an ability used to impress and be admired by others [38]; therefore, people with anxious attachment styles may be less prone to utilize it. Both benevolent humor and wit were found to negatively correlate with neuroticism, while extraversion was positively related to humor and wit [27]. Previous research described the negative relationship between extraversion and anxious attachment and the positive relationship with neuroticism [39,40].

Anxiety attachment was also positively related to irony, the comic style aimed at creating a mutual sense of superiority toward others by saying things that are different from what they mean and containing the opposite of what is meant and which insiders understand. These individuals may use irony to create a self-bond with people who value them and to distance others, thus maintaining their safety [38,41]. Moreover, the content of some of the irony items is more aimed toward in-group humor rather than just ironic statements. Given the consistent relationships between anxiety and self-defeating humor [28], irony may represent a way to say things at the speaker’s own expense to preserve relations with others.

Previous research has shown that avoidant attachment was negatively related to affiliative humor and anxious attachment was positively related to self-defeating humor [17]. In this research, when the avoidant attachment was considered, nonsense and sarcasm were found to be negatively associated to this attachment style. Avoidant people tend to be independent, and they do not rely on others for reassurance or emotional support. Their relationships will be unreliable and so they will dismiss or reject others. The avoidant attachment was related to an emotional regulatory style characterized by affect “minimization”, and these individuals are more hostile and defensive [8]. Individuals with a more prominent avoidance orientation are described as deactivating, distancing themselves from others when distressed, and less prone to search for social support [32,42]. As sarcasm is based on being critical of others, conveying contempt may be used to implement psychological and emotional distance from others [43]. Sarcasm may be a way to express one’s emotional distress without openly declaring what was intended to be safely protected by the use of a humorous attempt.

Nonsense is based on playing with incongruities and ridiculousness without any purpose, which contains incongruities that are only partly resolved, unresolved, or in which the apparent resolution adds yet another incongruity [44]. An avoidant attachment style is characterized by the continual inhibition of psychological and social relationship needs, leading to an avoidance of romantic relationships and emotional bonds [45]. In this regard, avoidance individuals may prefer nonsense as they would probably like to play with their own ideas rather than interact with people. Moreover, nonsense was negatively related to seriousness traits, showing how this comic style entails a cognitive component of habitually interpreting events as less serious and more playful [26]. We assume that avoidant individuals may use this form of humor to cope with stress.

Some limitations need to be acknowledged for consideration by future researchers. First, our data were confined to a particular geographic location, and the results will need to be replicated and confirmed in larger and different samples of other cultures. Second, we used entirely cross-sectional self-report data. Despite this limitation, these results pave the way for future studies aimed at exploring the causalities underlying these relationships. Third, self-report measures were used, and future studies with different assessment tools are required. Lastly, we measured the insecure attachment styles using a questionnaire based on romantic relationships. Future studies must consider assessing the other attachment styles and using different instruments, such as the one aimed at investigating relationships with parents. Moreover, exploring the distinctive patterns of use of comic styles in people with different marital statuses is a worthy and promising line of research.

In conclusion, this is the first study to focus on the relationships between the two insecure attachment styles and the eight comic styles, considering both lighter and darker forms of humor. The findings of the current study support the notion that individual differences in humor sense are related to the various attachment orientations. These findings may be of interest for both researchers and psychologists in their work with patients. From the point of view of how this may be applied in a therapeutic context, being aware of the patient’s humor may guide the focus of the interview, helping the therapist in the assessment of a patients.

## Figures and Tables

**Table 1:** Intercorrelations among the comic styles.

	Fun	Humor	Nonsense	Wit	Irony	Satire	Sarcasm	Cynicism
Fun	1							
Humor	0.37 ***	1						
Nonsense	0.42 ***	0.44 ***	1					
Wit	0.44 ***	0.60 ***	0.27 ***	1				
Irony	0.45 ***	0.38 ***	0.37 ***	0.54 ***	1			
Satire	0.45 ***	0.56 ***	0.40 ***	0.58 ***	0.58 ***	1		
Sarcasm	0.42 ***	0.31 ***	0.29 ***	0.47 ***	0.61 ***	0.60 ***	1	
Cynicism	0.31 ***	0.22 ***	0.35 ***	0.33 ***	0.53 ***	0.58 ***	0.63 ***	1

Note. N = 636; *** *p* < 0.001.

**Table 2:** Correlations among variables.

	Fun	Humor	Nonsense	Wit	Irony	Satire	Sarcasm	Cynicism
ECR_Anxiety	0.11 **	−0.07	−0.01	−0.02	0.14 ***	0.05	0.11 **	0.10 **
ECR_Avoidance	0.02	−0.02	0.12 **	0.01	0.11 **	0.06	0.13 ***	0.14 ***

Note. N = 636; ** *p* < 0.01; *** *p* < 0.001.

**Table 3:** Hierarchical regression of comic styles with ECR controlled for age and gender.

	Fun	Humor	Nonsense	Wit	Irony	Satire	Sarcasm	Cynicism	Δ R^2^
ECR_Anxiety	0.09	−0.12 *	−0.03	−0.11 *	0.14 *	0.07	0.02	−0.01	0.04 ***
ECR_Avoidance	−0.04	−0.08	0.10 *	−0.05	0.08	−0.07	0.11 *	0.09	0.04 ***

Note. N = 634. * p < 0.05; *** *p* < 0.001.

## Data Availability

The datasets generated during and/or analyzed during the current study are available from the corresponding author on reasonable request.

## References

[1] Mikulincer M., Shaver P.R., Vangelisti A.L., Perlman D. (2018). Attachment theory as a framework for studying relationship dynamics and functioning. The Cambridge Handbook of Personal Relationships.

[2] Bowlby J. (1982). Attachment and loss: Retrospect and prospect. Am. J. Orthopsychiatry.

[3] Bowlby J. (1973). Attachment and Loss: Volume II: Separation, Anxiety and Anger.

[4] Fraley R.C., Shaver P.R. (2000). Adult Romantic Attachment: Theoretical Developments, Emerging Controversies, and Unanswered Questions. Rev. Gen. Psychol..

[5] Bartholomew K., Horowitz L.M. (1991). Attachment styles among young adults: A test of a four-category model. J. Pers. Soc. Psychol..

[6] Grossmann K.E., Grossmann K., Zimmermann P., Cassidy J., Shaver P.R. (1999). A wider view of attachment and exploration: Stability and change during the years of immaturity. Handbook of Attachment: Theory, Research, and Clinical Applications.

[7] Main M., Solomon J., Greenberg M.T., Cicchetti D., Cummings E.M. (1990). Procedures for identifying infants as disorganized/disoriented during the Ainsworth Strange Situation. Attachment in the Preschool Years: Theory, Research, and Intervention.

[8] Mikulincer M., Florian V., Simpson J.A., Rholes W.S. (1998). The relationship between adult attachment styles and emotional and cognitive reactions to stressful events. Attachment Theory and Close Relationships.

[9] Mikulincer M., Shaver P.R. (2012). An attachment perspective on psychopathology. World Psychiatry.

[10] Howland M., Simpson J.A. (2013). Attachment orientations and reactivity to humor in a social support context. J. Soc. Pers. Relatsh..

[11] Feeney J., Fitzgerald J. (2019). Attachment, conflict and relationship quality: Laboratory-based and clinical insights. Curr. Opin. Psychol..

[12] Simpson J.A., Rholes W.S. (2019). Adult attachment orientations and well-being during the transition to parenthood. Curr. Opin. Psychol..

[13] Luevano V.X., Pablo J.N., Velazquez M.L., Chance B., Ramirez B. (2021). Attachment as a predictor of attraction to humor styles. Pers. Individ. Differ..

[14] Martin R.A., Ford T. (2018). The Psychology of Humor: An Integrative Approach.

[15] Dozois D.J.A., Martin R.A., Bieling P.J. (2009). Early Maladaptive Schemas and Adaptive/Maladaptive Styles of Humor. Cogn. Ther. Res..

[16] Poncy G.W. (2017). Maladaptive Humor Styles as Mediators of the Relationship between Attachment Insecurity and Emotion Regulation. Humor.

[17] Kazarian S.S., Martin R.A. (2004). Humour styles, personality, and well-being among Lebanese university students. Eur. J. Pers..

[18] Taher D., Kazarian S.S., Martin R.A. (2008). Validation of the Arabic Humor Styles Questionnaire in a Community Sample of Lebanese in Lebanon. J. Cross-Cult. Psychol..

[19] Cann A., Norman M.A., Welbourne J.L., Calhoun L.G. (2008). Attachment styles, conflict styles and humour styles: Interrelationships and associations with relationship satisfaction. Eur. J. Pers..

[20] Saroglou V., Scariot C. (2002). Humor Styles Questionnaire: Personality and educational correlates in Belgian high school and college students. Eur. J. Pers..

[21] Sar-El D., Mikulincer M., Doron G. (2013). Attachment Orientations and Individual Differences in Humor. J. Soc. Clin. Psychol..

[22] Miczo N., Averbeck J.M., Mariani T. (2009). Affiliative and Aggressive Humor, Attachment Dimensions, and Interaction Goals. Commun. Stud..

[23] Ford T.E., Lappi S.K., O’Connor E.C., Banos N.C. (2017). Manipulating humor styles: Engaging in self-enhancing humor reduces state anxiety. Humor.

[24] Leist A.K., Müller D. (2012). Humor Types Show Different Patterns of Self-Regulation, Self-Esteem, and Well-Being. J. Happiness Stud..

[25] Besser A., Luyten P., Mayes L.C. (2012). Adult attachment and distress: The mediating role of humor styles. Individ. Differ. Res..

[26] Heintz S. (2019). Locating eight comic styles in basic and broad concepts of humor: Findings from self-reports and behavior tests. Curr. Psychol..

[27] Ruch W., Heintz S., Platt T., Wagner L., Proyer R. (2018). Broadening Humor: Comic Styles Differentially Tap into Temperament, Character, and Ability. Front. Psychol..

[28] Heintz S., Ruch W. (2019). From four to nine styles: An update on individual differences in humor. Pers. Individ. Differ..

[29] Ruch W., Heintz S. (2016). The German version of the Humor Styles Questionnaire: Psychometric properties and overlap with other styles of humor. Eur. J. Psychol..

[30] Saroglou V., Lacour C., Demeure M.-E. (2010). Bad Humor, Bad Marriage: Humor Styles in Divorced and Married Couples. Eur. J. Psychol..

[31] Dionigi A., Duradoni M., Vagnoli L. (2021). Humor and personality: Psychometric properties of the Italian version of the comic styles markers and its relationships with the big five personality traits. Curr. Psychol..

[32] Wei M., Russell D.W., Mallinckrodt B., Vogel D.L. (2007). The Experiences in Close Relationship Scale (ECR)-Short Form: Reliability, Validity, and Factor Structure. J. Pers. Assess..

[33] Faul F., Erdfelder E., Lang A.-G., Buchner A. (2007). G*Power 3: A flexible statistical power analysis program for the social, behavioral, and biomedical sciences. Behav. Res. Methods.

[34] Faul F., Erdfelder E., Buchner A., Lang A.-G. (2009). Statistical power analyses using G*Power 3.1: Tests for correlation and regression analyses. Behav. Res. Methods.

[35] Hall J.A. (2013). Humor in Long-Term Romantic Relationships: The Association of General Humor Styles and Relationship-Specific Functions with Relationship Satisfaction. West. J. Commun..

[36] Candel O.-S., Turliuc M.N. (2019). Insecure attachment and relationship satisfaction: A meta-analysis of actor and partner associations. Pers. Individ. Differ..

[37] Kafetsios K., Nezlek J.B. (2002). Attachment styles in everyday social interaction. Eur. J. Soc. Psychol..

[38] Dionigi A., Duradoni M., Vagnoli L. (2022). Humor and the dark triad: Relationships among narcissism, Machiavellianism, psychopathy and comic styles. Pers. Individ. Differ..

[39] Noftle E.E., Shaver P.R. (2006). Attachment dimensions and the big five personality traits: Associations and comparative ability to predict relationship quality. J. Res. Pers..

[40] Shaver P.R., Brennan K.A. (1992). Attachment Styles and the “Big Five” Personality Traits: Their Connections with Each Other and with Romantic Relationship Outcomes. Personal. Soc. Psychol. Bull..

[41] Torres-Marín J., Navarro-Carrillo G., Carretero-Dios H. (2022). Differentiating the traits of the Dark Tetrad in their linkages with humor styles, dispositions toward ridicule and laughter, and comic styles. Pers. Individ. Differ..

[42] Mikulincer M., Shaver P.R., Pereg D. (2003). Attachment Theory and Affect Regulation: The Dynamics, Development, and Cognitive Consequences of Attachment-Related Strategies. Motiv. Emot..

[43] Winterheld H.A., Simpson J.A., Vangelisti A.L., Perlman D. (2018). Personality in close relationships. The Cambridge Handbook of Personal Relationships.

[44] Ruch W., McGhee P.E., Hehl F.-J. (1990). Age differences in the enjoyment of incongruity-resolution and nonsense humor during adulthood. Psychol. Aging.

[45] Mikulincer M., Shaver P.R. (2019). Attachment orientations and emotion regulation. Curr. Opin. Psychol..

